# A rare case of survival after traumatic blunt ventricular rupture

**DOI:** 10.1093/jscr/rjad059

**Published:** 2023-02-14

**Authors:** Ryan Yang, Michael Van Gent, Thomas Clements, Bryan Cotton, Michael Wandling

**Affiliations:** Department of General Surgery, McGovern Medical School, Houston, TX, USA; Department of Trauma/Surgical Critical Care, Red Duke Trauma Institute, Houston, TX, USA; Department of Trauma/Surgical Critical Care, Red Duke Trauma Institute, Houston, TX, USA; Department of Trauma/Surgical Critical Care, Red Duke Trauma Institute, Houston, TX, USA; Department of Trauma/Surgical Critical Care, Red Duke Trauma Institute, Houston, TX, USA

## Abstract

A male in his 40s presented to the trauma center via air ambulance after colliding with a cement wall at highway speeds. Cross-sectional imaging revealed a right ventricular pseudoaneurysm, confirmed by echocardiography. He was taken emergently to the operating room where he was found to have a pericardial laceration, hemopericardium and a right ventricular rupture, which was primarily repaired. Postoperatively, the patient was transferred to intensive care and after 34 days in the hospital was ultimately discharged home.

## INTRODUCTION

Blunt cardiac rupture (BCR) is an exceedingly rare sequelae of blunt chest trauma with incidence rates reported as low as 0.007–0.45% of patients that present alive to the hospital [1, 2]. In addition to rarity, BCR is an exceptionally morbid injury with 44% of patients either deceased in the prehospital setting or shortly after arrival. In-hospital mortality of surviving patients is as high as 45% [1]. Overall, the mortality of blunt cardiac rupture is as high as 80% [3]. To the extent of our knowledge, only 18 cases of survival have been reported since the 1950s [4, 5]. Despite the rare presentation, the exceptionally high mortality associated with blunt ventricular rupture warrants attention to ensure prompt recognition and management.

## CASE REPORT

**Figure 1:**
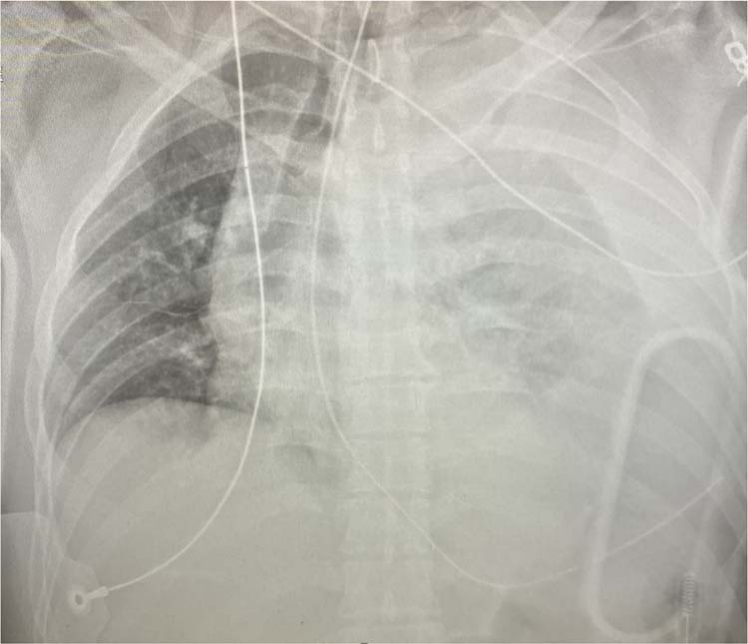
Chest x-ray demonstrating left hemothorax.

An approximately 40-year-old male was involved in a motor vehicle collision after hitting a concrete wall at highway speeds. The patient was found to have a Glasgow Coma Scale (GCS) score of 3 on scene, so he was intubated and brought to the trauma center as a level I trauma activation via helicopter. Upon arrival, his primary survey was significant for an airway secured with an endotracheal tube, tachycardic to the 110 s, hypotensive with a systolic in the 60s and GCS of 3 T. His secondary survey identified an open mandibular fracture, right hip dislocation and an obvious left thigh deformity with palpable distal pulses. His chest x-ray revealed a left hemothorax (see Fig. 1), his pelvic-x-ray confirmed the hip dislocation, and his Focused Assessment with Sonography for Trauma (FAST) examination was negative for free fluid in all four windows. A left thoracostomy tube was immediately placed, which evacuated 500 cc of blood with minimal continuous output. He was given 1 unit of packed red blood cells (PRBC) with blood pressure normalization but persistent tachycardia.

**Figure 2:**
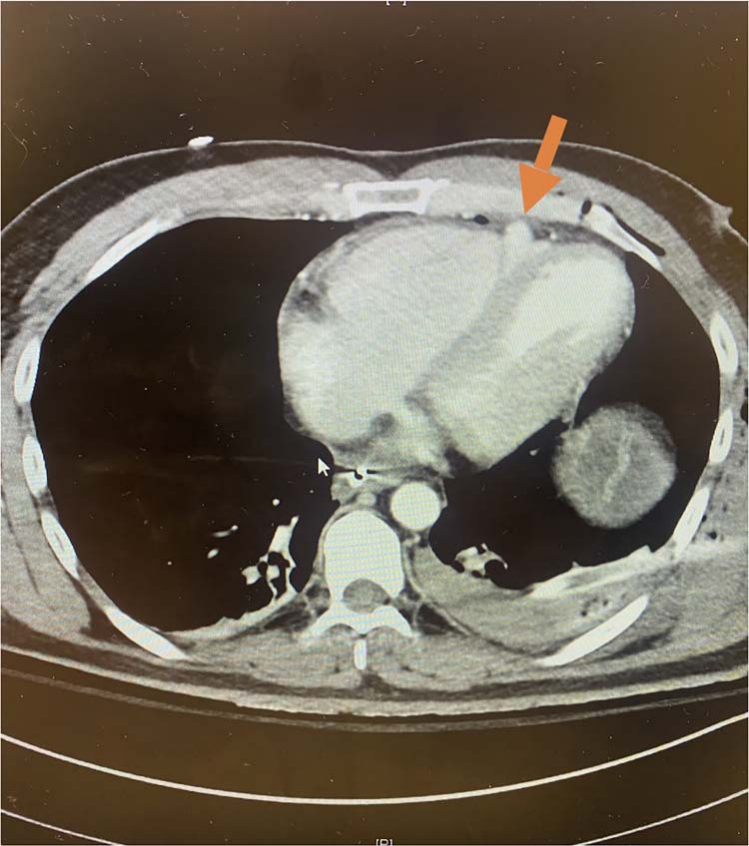
Axial CT imaging demonstrating pseudoaneurysm of right ventricular free wall rupture.

**Figure 3:**
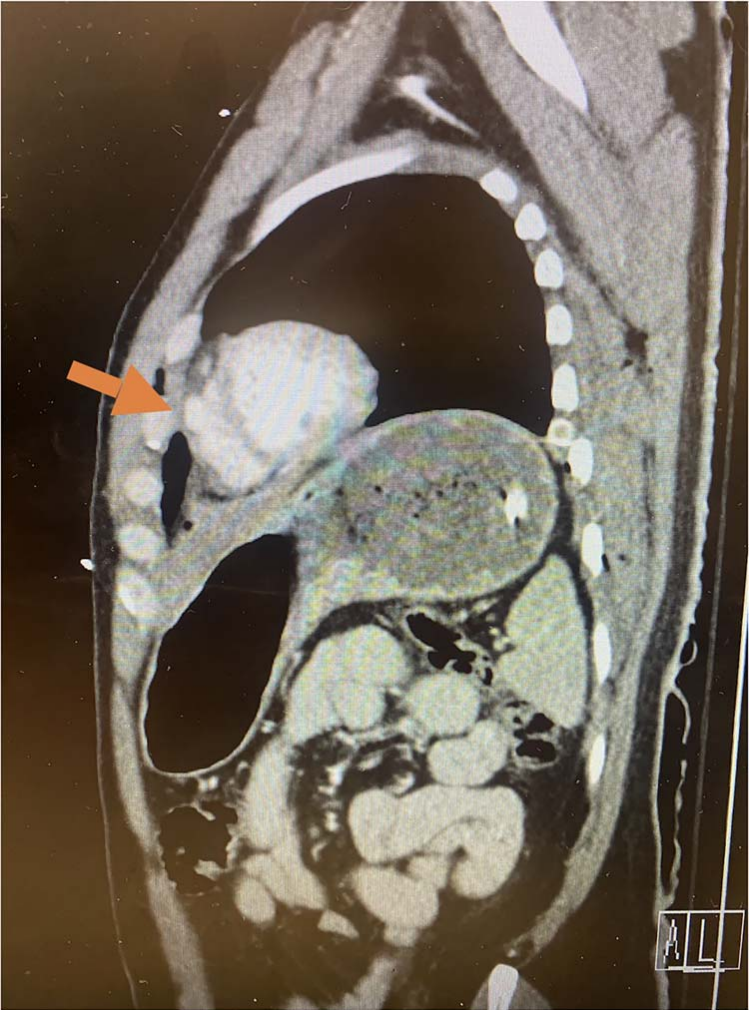
Sagittal CT imaging demonstrating pseudoaneurysm of right ventricular free wall rupture.

Given his hemodynamic normalization without ongoing transfusion requirements, he was taken to the computed tomography (CT) scanner for axial imaging of the head, chest, abdomen and pelvis. A CT angiogram of the chest revealed trace pneumopericardium and pneumomediastinum with trace hemopericardium. A 1.7 × 0.7 cm out-pouching at the anterior aspect of the right ventricular apex concerning for a traumatic pseudoaneurysm was also detected (see Figs 2 and 3). His other injuries were notable for subarachnoid and subdural hemorrhages, left temporal bone fracture, open mandibular fracture, left 3–6th, 10th and 11th rib fractures, a 4th lumbar vertebrae burst fracture with 1st and 3rd lumbar vertebrae compression fractures, 8–11th thoracic vertebrae compression fractures, and a left femoral neck fracture. He was subsequently transferred back to the trauma bay where a formal trans-thoracic echocardiogram was immediately performed, which demonstrated a dyskinetic area of the right ventricular apex with paradoxical out-pouching during systole—suggestive of pseudoaneurysm and confirming the diagnosis on axial imaging.

**Figure 4:**
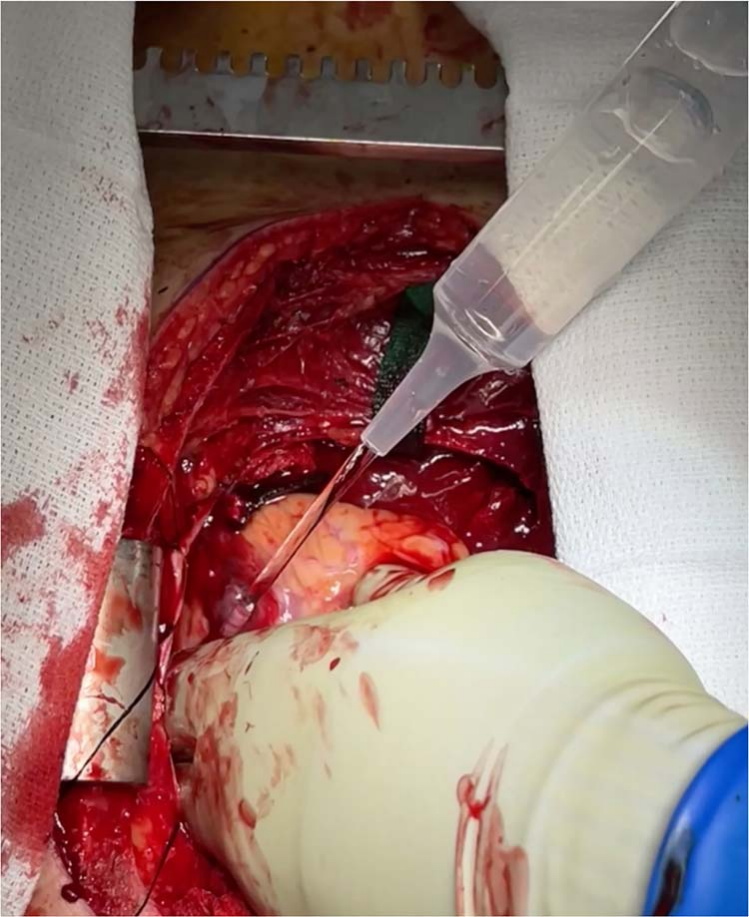
Intraoperative photo demonstrating extravasation from ventricular rupture.

**Figure 5:**
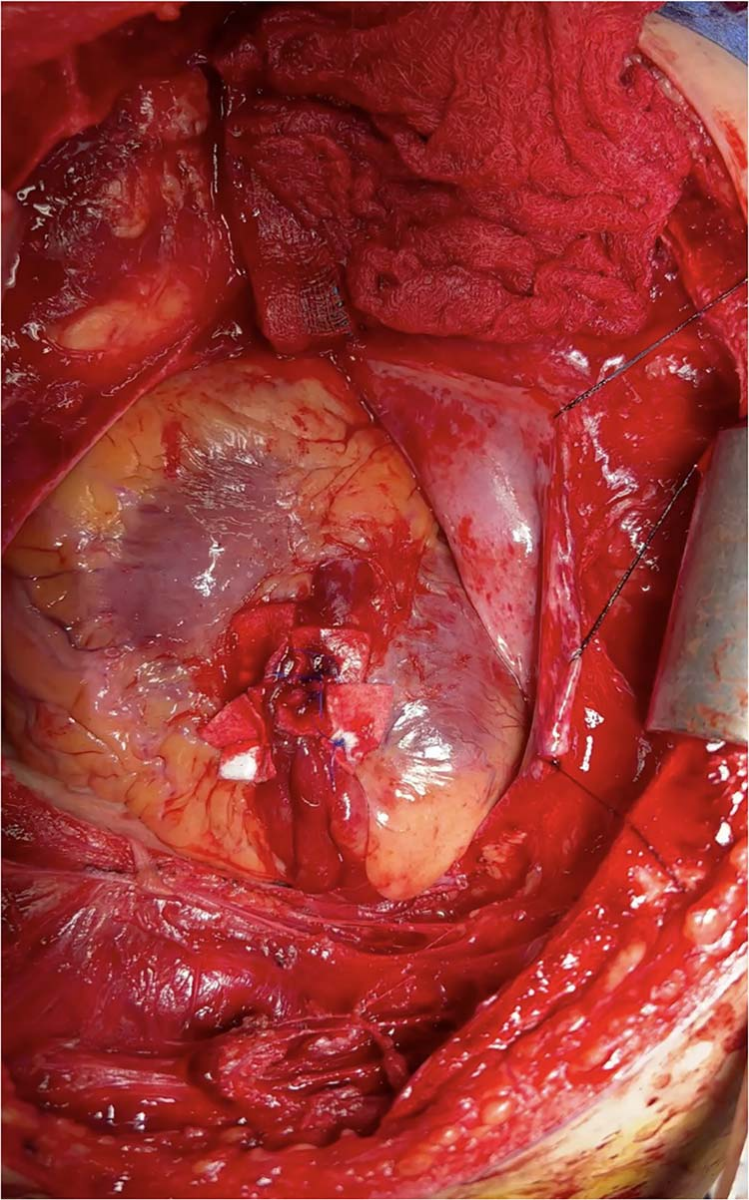
Intraoperative photo demonstrating pledgeted horizontal mattress repair of ventricular rupture.

At this time, the trauma surgeons decided to take the patient emergently to the operating room. We performed a median sternotomy and discovered a 4-cm laceration of the pericardium (see Fig. 4) and left mediastinal pleura, moderate hemopericardium with thrombus, and 3-cm rupture of the right ventricle adjacent to the left anterior descending artery, consistent with the CT scan. Approximately, 2 cm of this injury was full-thickness and the remaining 1 cm was partial thickness involving most, but not the entirety, of the ventricular wall (see Supplementary Intra-Operative Video). The right ventricular injury was repaired primarily using pledgeted 3-0 prolene horizontal mattress sutures (see Fig. 5). The ventricle was hemostatic and noted to have good contractility. At this time, we performed a diagnostic laparoscopy to rule out a possible left diaphragmatic injury noted on CT. A grade I segment VII hepatic laceration was identified that was managed laparoscopically with hepatorrhaphy using electrocautery.

Post-operatively, he was transferred to the Surgical Trauma Intensive Care Unit (STICU). His 34-day hospital included a percutaneous tracheostomy and gastrostomy, and orthopedic fixation. He was ultimately discharged with a GCS of 15 and home physical therapy services.

## DISCUSSION

Traumatic blunt cardiac rupture (BCR) is rare following blunt mechanisms of injury with exceptionally high mortality and few reported cases. Of those reported, the majority (70%) sustained BCR during a high-velocity MVC. The theorized explanation for the etiology of BCR is transduction of the collision’s kinetic energy with resultant sudden pressure elevations in the cardiac chambers. This is attributed to direct thoracic compression, which is supported by the frequent association of rib fractures in these patients with polytraumatic injuries, or indirectly caused by rapid venous return resulting from intra-abdominal vena cava compression from the abdominal portion of the seat belt. This can result in cardiac tamponade from a ruptured myocardium [6] or conversely, a herniation of the heart through a pericardial or mediastinal pleural rupture and subsequent torsion of the great vessels [7]_._

Prompt diagnosis of ventricular rupture is of vital importance for any meaningful life-saving opportunity. Typically, the FAST examination is an effective adjunct for early identification of pericardial effusion and the sentinel sign that a patient may need operative intervention for a cardiac injury. However, FAST examination can miss cardiac injuries in the setting of concomitant injury to the mediastinal pleura due to pericardial decompression into the pleural space [8–10]. Given the high mortality in the setting of frequent false negative pericardial window on FAST, especially in the setting of a left hemothorax, providers responding to patients with blunt chest trauma should maintain a high index of suspicion for potentially fatal underlying cardiac injury.

When blunt cardiac rupture is confirmed or suspected on cross-sectional imaging or on transthoracic echocardiogram, surgeons should immediately take the patient to the operating room for mediastinal exploration and definitive surgical repair. Our patient presented in hemorrhagic shock—likely due to concomitant ruptures of the ventricular wall, pericardium, and mediastinal pleural preventing tamponade and permitting exsanguination into his thorax. This massive blood loss and associated hypotension is likely what allowed him to form the clot overlying the rupture that we discovered intra-operatively. Had the trauma surgeons not recognized this injury and repaired the rupture, the patient would have been at high risk of subsequent mechanical disruption of the clot and fatal exsanguination—either upon attaining euvolemia with resuscitation and restoring normotension to the cardiac chambers or with increased thoracic pressure from even a cough.

While ventricular rupture is both a rare and devastating injury in the setting of blunt chest trauma, there are several reports in the literature of survivable cases discussed herein. We submit that early diagnosis and timely surgical repair should be heavily considered to optimize survival in the setting of these highly mortal injuries.

## CONFLICT OF INTEREST STATEMENT

None declared.

## FUNDING

None.

## Supplementary Material

Intraoperative_Video_rjad059
